# mGluR5 mediates post-radiotherapy fatigue development in cancer patients

**DOI:** 10.1038/s41398-018-0161-3

**Published:** 2018-05-30

**Authors:** Li Rebekah Feng, Juan Luis Fernández-Martínez, Kristien J.M. Zaal, Enrique J deAndrés-Galiana, Brian S. Wolff, Leorey N. Saligan

**Affiliations:** 1 National Institute of Nursing Research, National Institutes of Health, Bethesda, MD USA; 2 Universidad de Oviedo, Oviedo, Spain; 3 Light Imaging Section, National Institute of Arthritis and Musculoskeletal and Skin Diseases, National Institutes of Health, Bethesda, MD USA; 4 National Institute of Nursing Research/National Cancer Institute, National Institutes of Health, Bethesda, MD USA

## Abstract

Cancer-related fatigue (CRF) is a common burden in cancer patients and little is known about its underlying mechanism. The primary aim of this study was to identify gene signatures predictive of post-radiotherapy fatigue in prostate cancer patients. We employed Fisher Linear Discriminant Analysis (LDA) to identify predictive genes using whole genome microarray data from 36 men with prostate cancer. Ingenuity Pathway Analysis was used to determine functional networks of the predictive genes. Functional validation was performed using a T lymphocyte cell line, Jurkat E6.1. Cells were pretreated with metabotropic glutamate receptor 5 (mGluR5) agonist (DHPG), antagonist (MPEP), or control (PBS) for 20 min before irradiation at 8 Gy in a Mark-1 γ-irradiator. NF-κB activation was assessed using a NF-κB/Jurkat/GFP Transcriptional Reporter Cell Line. LDA achieved 83.3% accuracy in predicting post-radiotherapy fatigue. “Glutamate receptor signaling” was the most significant (*p* = 0.0002) pathway among the predictive genes. Functional validation using Jurkat cells revealed clustering of mGluR5 receptors as well as increased regulated on activation, normal T cell expressed and secreted (RANTES) production post irradiation in cells pretreated with DHPG, whereas inhibition of mGluR5 activity with MPEP decreased RANTES concentration after irradiation. DHPG pretreatment amplified irradiation-induced NF-κB activation suggesting a role of mGluR5 in modulating T cell activation after irradiation. These results suggest that mGluR5 signaling in T cells may play a key role in the development of chronic inflammation resulting in fatigue and contribute to individual differences in immune responses to radiation. Moreover, modulating mGluR5 provides a novel therapeutic option to treat CRF.

## Introduction

Persistent fatigue is a debilitating condition that affects up to 80% of cancer patients^1^. It is not uncommon for fatigue to persist long after cancer treatment, and to negatively impact the quality of life in these patients^1,2^. Although the underlying mechanisms of persistent fatigue remain elusive, emerging evidence suggests that unresolved inflammation after cancer treatment plays a role in the chronicity of cancer fatigue^3–7^.

Radiotherapy is a highly effective standard of care treatment for many types of cancer^8^. However, post-treatment complications such as fibrosis and inflammation often occur as a result of repeated stress^9^. Reactive oxygen species (ROS) produced by ionizing radiation can lead to point mutations in mitochondrial and genomic DNA, mitochondrial dysfunction, genomic instability, oxidative stress, release of pro-inflammatory cytokines, and a prolonged inflammatory state^9–11^. Subsequently, unresolved inflammation triggered by repeated stress has been shown to result in “sickness behavior,” a cluster of symptoms including fatigue, depression, and increased sensitivity to pain^5,12,13^.

The association between inflammation and fatigue after repeated stress from daily exposure of ionizing radiation warrants further investigation to determine causality. It is possible that transcriptional activation of nuclear factor kappa B (NF-κB), which is a ubiquitous transcription factor that regulates expression of various genes including those involved in inflammation^14^, may provide the missing link. In fact, NF-κB activation has been found in cancer and after exposure to ionizing radiation^15^. It is the most studied transcription factor because of its central role in regulating the expression of various pro-inflammatory cytokines^11^.

Our aim was to explore novel gene signatures that can identify cancer patients who are at risk for developing post-radiotherapy fatigue. We have previously shown that worsening of fatigue 1–2 years post-radiotherapy was related to chronic inflammation^6^. In this study, we investigated genetic vulnerabilities that lead to fatigue development, as well as underlying mechanisms of chronic inflammation in fatigued patients. We hypothesized that aberrant activation of NF-κB and pathological T cell activation would help explain the development of fatigue following repeated exposure to radiotherapy in at-risk individuals.

## Materials and methods

### Participants

The current study (NCT00852111) was approved by the Institutional Review Board (IRB) of the National Institutes of Health (NIH), Bethesda, Maryland. All participants enrolled in this study were euthymic men, 18 years of age or older, who were diagnosed with non-metastatic prostate cancer with or without prior prostatectomy, and were scheduled to receive external beam radiation therapy (EBRT). The entire EBRT treatment lasted 38–42 days, depending on the clinical stage of the prostate disease. Potential participants were excluded if they had a progressive disease that could cause significant fatigue, had psychiatric disease within the past five years, had uncorrected hypothyroidism or anemia, or had a second malignancy. Individuals who used sedatives, steroids, or non-steroidal anti-inflammatory agents were also excluded. Participants were recruited from September 2009 to November 2014 at the Magnuson Clinical Research Center at the NIH. Signed written informed consents were obtained prior to study participation.

### Instruments

Prostate specific antigen (PSA) and C-reactive protein (CRP) were measured from plasma samples sent to NIH Clinical Center, Department of Laboratory Medicine for routine laboratory analyses. Fatigue, as the primary outcome measure, was assessed in all participants using the 13-item Functional Assessment of Cancer Therapy-Fatigue (FACT-F), which is a frequently used, validated, reliable, stand-alone measure of fatigue in cancer therapy (coefficient alpha = 0.95–0.96)^16^. Each item response is rated on a 0–4 scale, where a 0 represents “not at all” and a 4 indicates that the respondent relates to the corresponding statement “very much.” Total scores range from 16–53 with lower scores reflecting high fatigue intensity. Subjects were considered to be fatigued when there was a clinically significant decrease (worsening of fatigue symptom) in FACT-F score of ≥3 points from baseline (prior to radiotherapy initiation) to 1-year post radiotherapy^17^.

### Gene expression profile by microarray and RT-qPCR validation

RNA extraction and Affymetrix microarray chips (HG U133 Plus 2.0, Santa Clara, CA) were processed as previously described^6,18^. Affymetrix GeneChip Command Console (AGCC, 3.0 V) was used to scan images during data acquisition. Affymetrix CEL files (GEO accession GSE30174) containing raw intensity data were imported into Partek Genomics Suite 6.6 (Partek Inc., St. Louis, MO), log transformed, and normalized using the robust multi-array average (RMA) algorithm. Partek batch removal analysis of variance (ANOVA) was used to eliminate differences due to batch variation. ANOVA with false discovery rate (FDR) correction was used to identify differentially expressed genes (FDR <5%). The gene of interest was further confirmed with a TaqMan-based real-time quantitative PCR (RT-qPCR) using gene-specific primers (Thermo Fisher Scientific, Waltham, MA). Following genomic DNA elimination, a first-strand RNA-cDNA PCR template was generated from 150 ng of RNA using the High-capacity cDNA Reverse Transcription Kit (Thermo Fisher Scientific). RT-qPCR was performed on a QuantStudio 6 Flex instrument (Thermo Fisher Scientific), and the gene of interest was normalized to GAPDH endogenous control.

### Machine learning methodologies

**Fig. 1:**
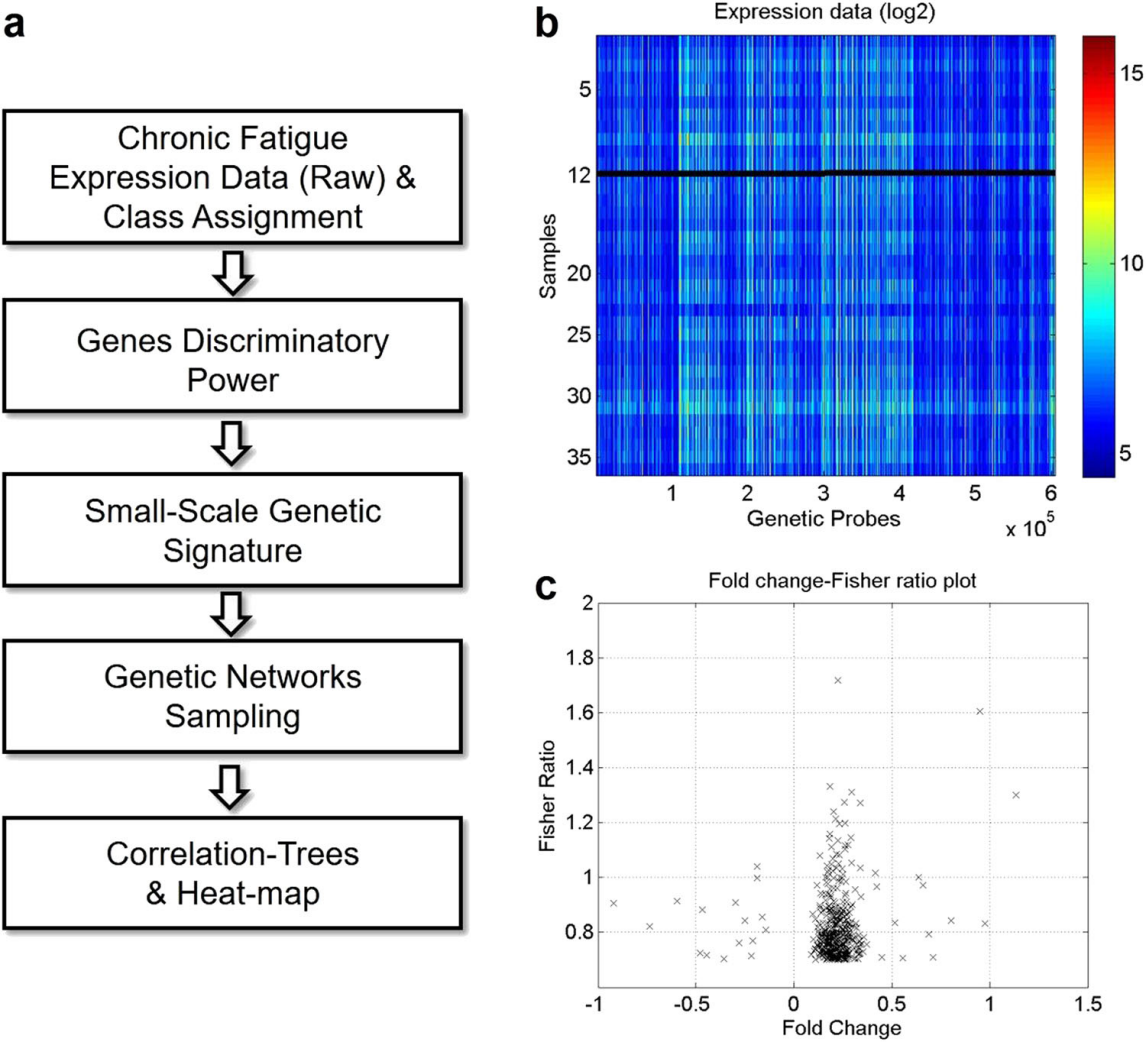
Flow chart of the machine learning methodology. **a** Flow chart of the machine learning methodology. **b** Microarray dataset visualized as log_2_. A total of 420 genes with Fisher’s ratio between [0.70, 1.72] and fold change between [−0.92, −0.15] and [0.09, 1.13] were selected using the feature selection procedure. **c** Fisher’s ratio-fold change plot demonstrates that most differentially expressed genes were different from those with the greatest Fisher’s ratio

The machine learning methodology used to identify the most discriminatory genes is described in Fig. 1a. Feature reduction and classification, as well as biomedical robots for fatigue phenotype classification, were applied to address the intrinsic uncertainty involved in the study^19^. Briefly, for a given gene *j* in a two-class problem, *c*_1_*c*_2_:$FR_j(c_1,c_2) = \frac{{\left( {\mu _{j1} - \mu _{j2}} \right)^2}}{{\sigma _{j1}^2 + \sigma _{j2}^2}},$

$\mu _{j1}^{},\,\mu _{j2}^{}$ are measures of the center of distribution (means) of prognostic variables *j* in classes 1 and 2. $\sigma _{j1}^2,\sigma _{j2}^2$ are measures of the dispersion or variance within these classes. This method identifies genes that separate the classes furthest apart and are homogeneous within classes with low intra-class variance. The most discriminatory variables were determined and ranked in decreasing order by discriminatory power. The algorithm then identified minimum-size genetic signatures by recursive elimination of lower discriminatory genes to optimize leave-one-out-cross-validation (LOOCV) predictive accuracy. This method was based on the rationale that highly discriminatory genes span main features of the classification, whereas genes with lowest discriminatory power account for details in phenotype discrimination. LOOCV involves using a single sample from the original dataset as the validation data (sample test), and the remaining samples as training data to build the classifier. The class assignment was based on a nearest-neighbor classifier in the reduced base. Cross-validation was performed to determine predictive accuracy of the classifier for new samples with unknown class assignment. A random sampler was used to identify other networks of highly discriminatory genes. The most discriminatory gene networks after sampling were determined, and posterior sampling frequencies of genes involved in these networks were analyzed. Pathway analysis of the most discriminatory genes were analyzed using the “Core Analysis” function included in Ingenuity Pathway Analysis (IPA, Qiagen, Redwood City, CA).

### Immunocytochemistry and fluorescence quantification

Jurkat, Clone E6–1 (T lymphocyte cell line, ATCC® TIB-152™) cells were directly purchased from American Type Culture Collection (ATCC, Manassas, VA), authenticated by Short Tandem Repeat (STR) profiling and tested mycoplasma free by ATCC. Jurkat cells were cultured based on standard ATCC protocols. Jurkat cells were pretreated with either control (PBS), 200 μM DHPG (3,5-Dihydroxyphenylglycine hydrate, Sigma-Aldrich, St. Louis, MO), or 20 μM MPEP (6-Methyl-2-phenylethynyl pyridine hydrochloride, Sigma-Aldrich) 20 min before irradiation at 8 Gy in a Mark-1 γ-irradiator (JL Shepherd & Associates, San Fernando, CA). Twenty-four hours after irradiation or sham treatment, cells were rinsed with PBS and fixed in 4% paraformaldehyde and probed with an anti-metabotropic glutamate receptor 5 antibody [EPR2425Y] (mGluR5, catalog number: ab76316; Abcam, Cambridge, MA). Antibody:antigen complexes were visualized with Alexa Fluor 568-conjugated goat anti-rabbit secondary antibody (catalog number: A-11011; Thermo Fisher Scientific, Waltham, MA). Cell morphology was visualized with Alexa Fluor 488 Phalloidin (catalog number: A12379; Thermo Fisher Scientific). Nuclei were stained with DAPI (4′,6′-diamidino-2 phenylindole; catalog number: D1306; Thermo Fisher Scientific).

Immunofluorescent images are maximum projections of optical slices (0.7 micron spacing) collected on a Leica Biosystems SP5 confocal microscope (Leica Microsystems Inc., Buffalo Grove, IL), using a 40 × 1.25 NA oil objective. Alexa Fluor 488 Phalloidin was excited at 488 nm and the emission collected was from 493 to 558 nm. mGluR5-Alexa Fluor 568 was excited at 561 nm and the collect emission range was 568–700 nm. DAPI was exited at 405 nm and the collected emission was 410–478 nm. Images of 1024 by 1024 pixels (averaging of 4) were collected sequentially for each color using the confocal microscope.

NF-κB/Jurkat/GFP Transcriptional Reporter Cell Line, a clonal Jurkat cell line with stably integrated lentiviral transcriptional reporter vector with 30-fold NF-κB-dependent activation of GFP reporter gene, was purchased from System Biosciences (SBI, Palo Alto, CA) and cultured based on standard ATCC protocols. NF-κB/Jurkat/GFP Transcriptional Reporter Cell Line was treated as described above. Positive controls were treated with recombinant TNFα (PeproTech, Rocky Hill, NJ). GFP intensity, an indicator for NF-κB activation, was directly quantified in each well of a 96-well microplate using a fluorescence plate reader (Cytation 5 Cell Imaging Multi-Mode Reader, BioTek, Winooski, VT).

### Western blot analysis

Cells were collected 24 h after irradiation, washed with PBS, and lysed on ice in modified RIPA buffer (50 mm Tris–HCl pH 7.4, 1% NP-40, 0.25% sodium deoxycholate, 150 mm NaCl) supplemented with protease inhibitor cocktail (Sigma-Aldrich), using a hand-held homogenizer. Lysates were centrifuged at 17,000×*g* 15 min at 4 °C. Supernatants were retained as the soluble lysate, boiled for 5 min in the presence of Laemmli Sample Buffer (Bio-Rad Laboratories, Hercules, CA). All protein samples were subjected to denaturing SDS–polyacrylamide gradient gel electrophoresis followed by transfer to polyvinylidene difluoride (PVDF) membranes using the Trans-Blot Turbo Transfer System (Bio-Rad Laboratories) and Western blot analysis for mGluR5 [EPR2425Y] (catalog number: ab76316; Abcam). Membranes were re-probed with a primary antibody against GAPDH (catalog number: ab9485; Abcam) as a loading control (*n* = 3 for each condition). Immunoreactive complexes were visualized using Super Signal West Pico Chemiluminescent Substrate (Thermo Fisher Scientific), imaged and quantified using the ChemiDoc MP Imaging Systems (Bio-Rad Laboratories).

### Statistical analysis

Descriptive analyses were used to describe demographic characteristics of the sample. All data were expressed as mean ± s.e.m. Normal distribution of data was confirmed using the Shapiro–Wilk test. Using power analysis with an alpha of 0.05 and power of 80%, the projected sample size needed is approximately 13 subjects per group and a total of 26 subjects. False Discovery Rate (FDR) adjustment using the Benjamini-Hochberg procedure was applied for analyses of whole transcriptome microarray and the subsequent PCR validation. One-way analysis of variance (ANOVA) was used to determine significant differences in comparisons involving more than two groups, followed by post hoc non-directional two-tailed *t*-test with Bonferroni correction for between group comparisons (for analyses of Western Blot optical density, GFP intensity measurement, and RANTES concentrations). In vitro experiments were performed with an *n* = 8 per treatment group and repeated at least three times. *p*-values < 0.05 were considered significant. Statistical analyses were performed with SPSS statistics software version 23 (IBM SPSS, Purchase, NY).

## Results

### Gene signatures associated with fatigue intensification at 1-year post EBRT

Of the 36 subjects recruited to generate the predictive algorithm, 33.3% of subjects experienced significantly worsened fatigue at 1-year post radiotherapy, defined as a decrease in FACT-F score of 3 points or greater from baseline to 1-year post radiotherapy. We did not observe any significant differences in clinical characteristics between the two groups for age, body mass index (BMI), Gleason scores, T stage, or PSA levels (Table 1). The acute inflammatory marker, CRP, was significantly elevated (*p* = 0.02) in fatigued subjects (5.2 ± 2.8 mg/ml) compared to non-fatigued controls (1.3 ± 0.1 mg/ml) at baseline, but did not differ (*p* = 0.21) 1-year post radiation therapy (Table 1). Fatigue severity as measured by FACT-F 1-year post radiotherapy correlated significantly with baseline CRP (*r* = −0.34, *p* = 0.04), but not with CRP levels 1-year post radiotherapy (*r* = 0.05, *p* = 0.75).

**Table 1:** Demographics and clinical characteristics of sample population

	Total (*n* = 36)	Fatigued (*n* = 12)	Non-fatigue (*n* = 24)
Age (years)	66 ± 7.07	64 ± 7.50	67 ± 7.00
BMI	30 ± 4	31 ± 5.42	29.26 ± 3.75
Race			
Asian	5.56%	8.33%	4.17%
Black	19.44%	16.67%	20.83%
Hispanic	5.56%	8.33%	4.17%
White	69.44%	66.67%	70.83%
Ethnicity			
Hispanic/Latino	5.56%	8.33%	4.17%
Not Hispanic/Latino	83.33%	83.33%	83.33%
No Answer	11.11%	8.33%	12.50%
Education			
Less than high school	2.78%	0%	4.17%
9–12th, not a graduate	8.33%	16.67%	4.17%
High school grad/GED	8.33%	8.33%	8.33%
Associate degree/some college	5.56%	16.67%	0%
Bachelor’s degree	36.11%	41.67%	33.33%
Advanced degree	11.11%	0%	16.67%
No answer	27.78%	16.67%	33.33%
T-stage			
T1c	27.78%	16.67%	33.33%
T2, NOS	2.78%	0%	4.17%
T2a	27.78%	33.33%	25.00%
T2b	8.33%	16.67%	4.17%
T2c	16.67%	0%	25.00%
T3a	5.56%	16.67%	0%
Other	5.56%	8.33%	4.17%
Gleason score			
6	11.11%	8.33%	12.50%
7	44.44%	33.33%	50.00%
6	25.00%	33.33%	40.83%
9	19.44%	25.00%	16.67%
PSA baseline (ng/ml)	7.58 ± 17.59	11.19 ± 29.67	5.78 ± 6.29
PSA completion of EBRT (ng/ml)	0.48 ± 1.08	0.531 ± 1.38	0.46 ± 0.93
CRP baseline (mg/ml)	2.34 ± 0.77	5.24 ± 2.80	1.26 ± 0.18
CRP 1 yr post-EBRT (mg/ml)	2.45 ± 0.38	1.74 ± 1.40	2.78 ± 3.25

*BMI* body mass index, *EBRT* external beam radiation therapy, *ADT* androgen deprivation therapy, *PSA* prostate specific antigen, *CRP* C-reactive protein

Whole genome transcriptome microarray data used to identify the most discriminatory genes were visualized with the heat map shown in log_2_ (Fig. 1b). The feature selection procedure generated 420 genes with Fisher’s ratio between [0.70, 1.72] and fold change between [−0.92, −0.15] [0.09, 1.13] that were most predictive of fatigue intensification at 1-year post radiotherapy (Fig. 1b; complete list of the 420 genes is included in Supplementary Table 1). Predictive genes with the highest fold change were different from ones with the highest Fisher’s ratio (Fig. 1c).

### Metabotropic glutamate receptor 5 and fatigue

**Fig. 2:**
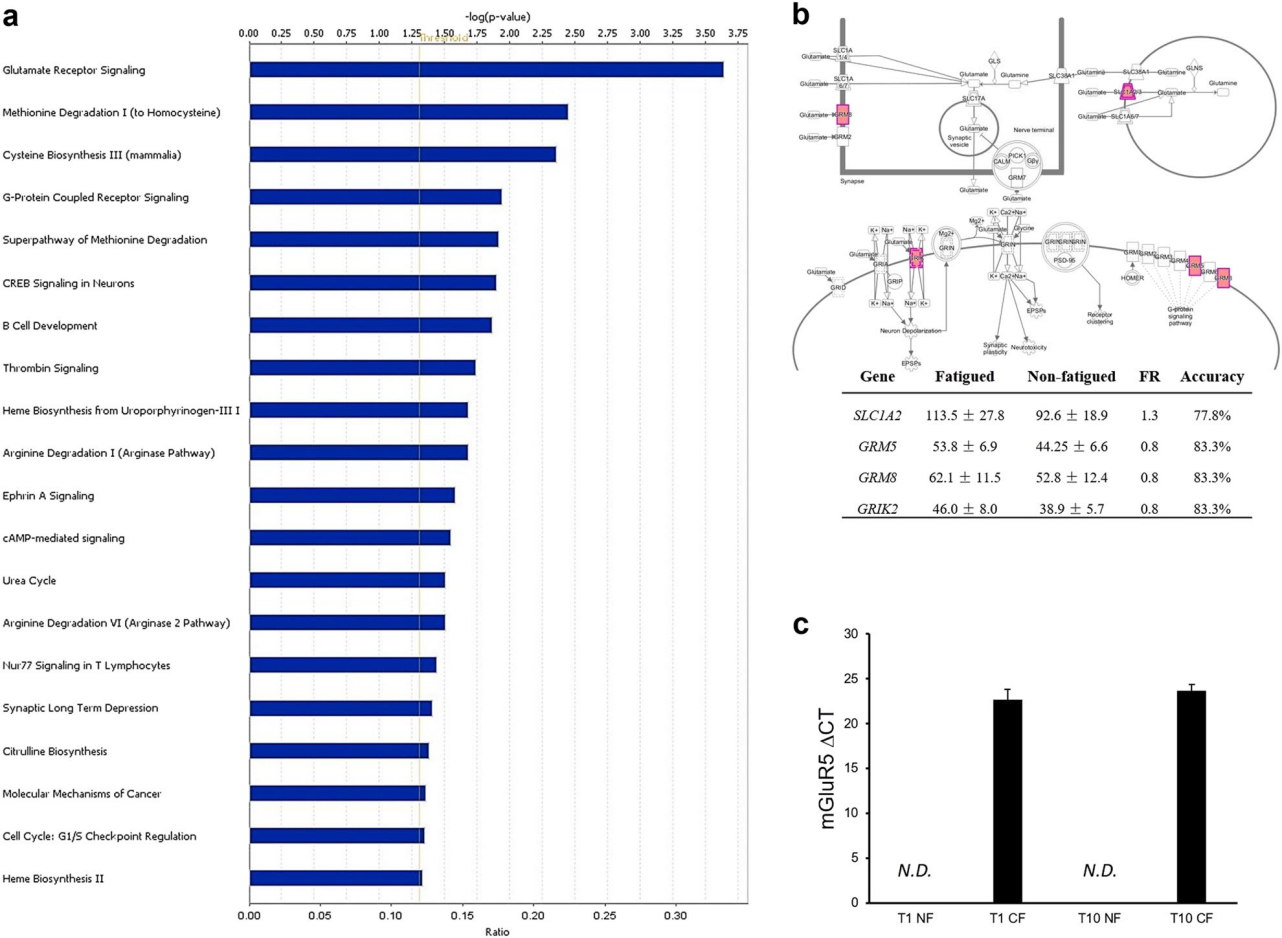
Gene signatures predictive of fatigue 1-year post EBRT. **a** Top canonical pathways of the predictive genes. Values are expressed as −log (*p*-value) indicating the significance of the enrichment of the predictive genes in each pathway. **b** *GRM5* encoding metabotropic glutamate receptor 5 (mGluR5) was among the genes with the highest predictive accuracy for fatigue 1-year post EBRT. Genes involved in glutamate signaling were found to be most predictive of fatigue 1-year post radiotherapy. **c** qRT-PCR validation demonstrating that *GRM5* was only detectable in fatigued subjects. ND, not detected. Error bars = s.e.m

Pathway analysis was performed to identify the top canonical pathways associated with the most discriminatory genes predictive of fatigue post-radiotherapy. Canonical pathways were ranked by significance (Fig. 2a). “Glutamate receptor signaling” was identified as the most significant canonical pathway (FDR corrected *p* = 1.47 × 10^−7^). “Glutamate receptor signaling” pathway genes that are predictive of fatigue 1-year post radiotherapy are shown in Fig. 2b. Within this pathway, *GRM5* encoding mGluR5 has a Fisher’s ratio of 0.8 and prediction accuracy at 83.3%. Using RT-qPCR, we confirmed that *GRM5* at a detection threshold cut off at 35 cycles was detectable in fatigued subjects, but not in non-fatigued subjects both at baseline and at 1-year post radiotherapy (Fig. 2c).

### mGluR5 activity affects T cell response to irradiation

**Fig. 3:**
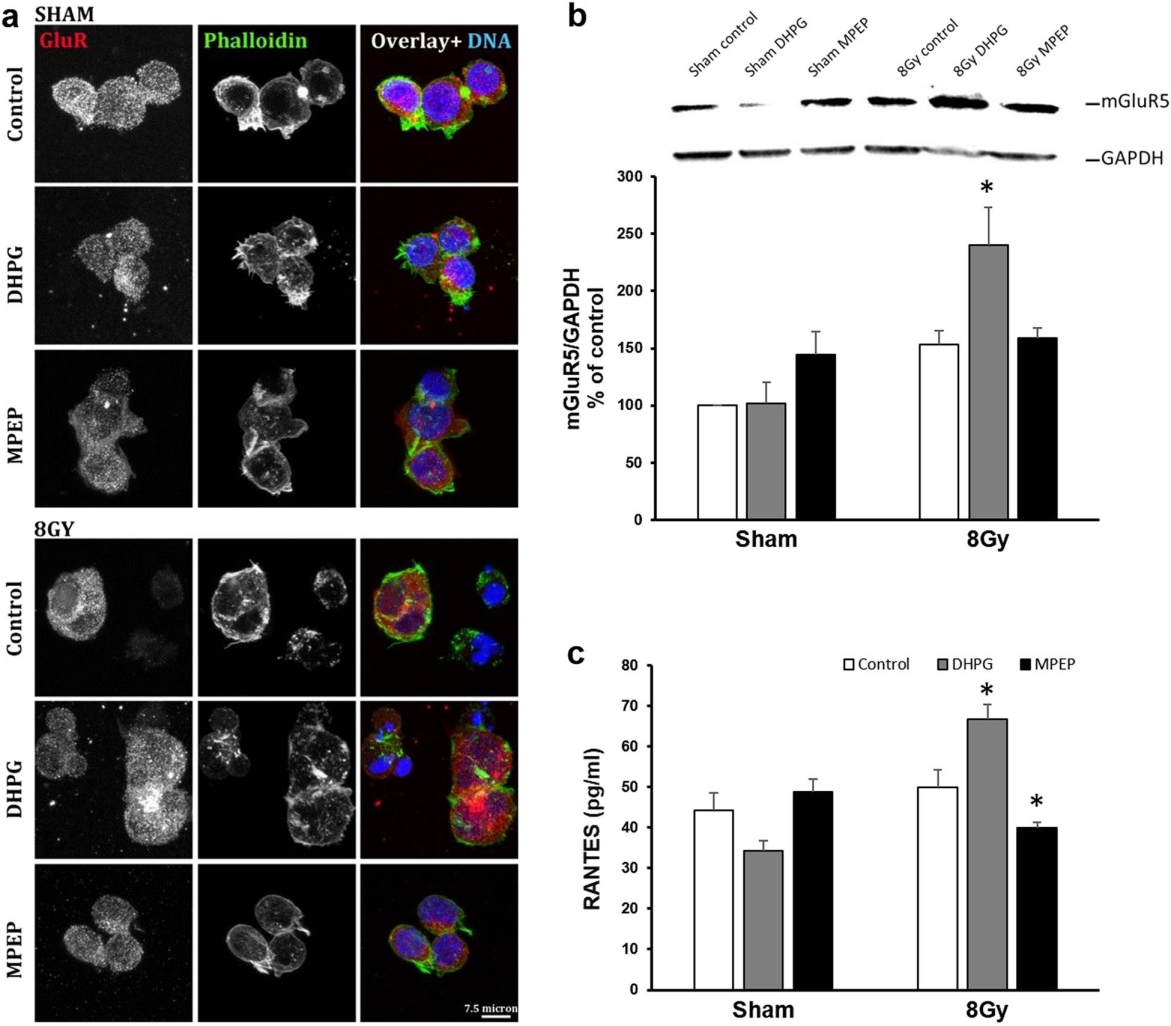
mGluR5 activity enhances effects of irradiation on T cell activation. **a** Confocal microscopy images of sham and irradiated Jurkat cells treated with control, DHPG, or MPEP. Red: mGluR5, Green: Phalloidin, Blue: DAPI. Scale bar = 7.5 μm. **b** Western blot data showing increased mGluR5 intensity in DHPG-treated irradiated Jurkat cells (*p* = 0.002). **c** DHPG pretreatment before radiation increased RANTES release into the culture media (*p* = 0.02), whereas MPEP decreased RANTES production (*p* = 0.001). Error bars = s.e.m. * *p* < 0.05

To investigate the role of mGluR5 in fatigue development, we utilized an in vitro model of T lymphocytes, Jurkat cells. Confocal images demonstrated that mGluR5 was expressed constitutively both intracellularly and on the cell membrane (Fig. 3a). Interestingly, clusters of mGluR5 were most prominent in Jurkat cells irradiated at 8 Gy pretreated with group I mGluR agonist DHPG (Fig. 3a, 8 Gy, bottom panel). Treatment with either DHPG or mGluR5 antagonist MPEP without irradiation did not change mGluR5 distribution (Fig. 3a, sham, top panel). Western blot analysis using whole cell protein lysates revealed that mGluR5 protein levels increased in the DHPG-treated cells irradiated at 8 Gy (Fig. 3b). RANTES (regulated on activation, normal T cell expressed and secreted), a chemokine released by activated T cells, increased in cell culture media collected from DHPG-treated irradiated cells (*p* = 0.02), whereas blocking mGluR5 with MPEP prior to irradiation decreased RANTES release (*p* = 0.0001) into the culture media (Fig. 3c).

### mGluR5 agonist treatment enhances irradiation-induced NF-kB activation

**Fig. 4:**
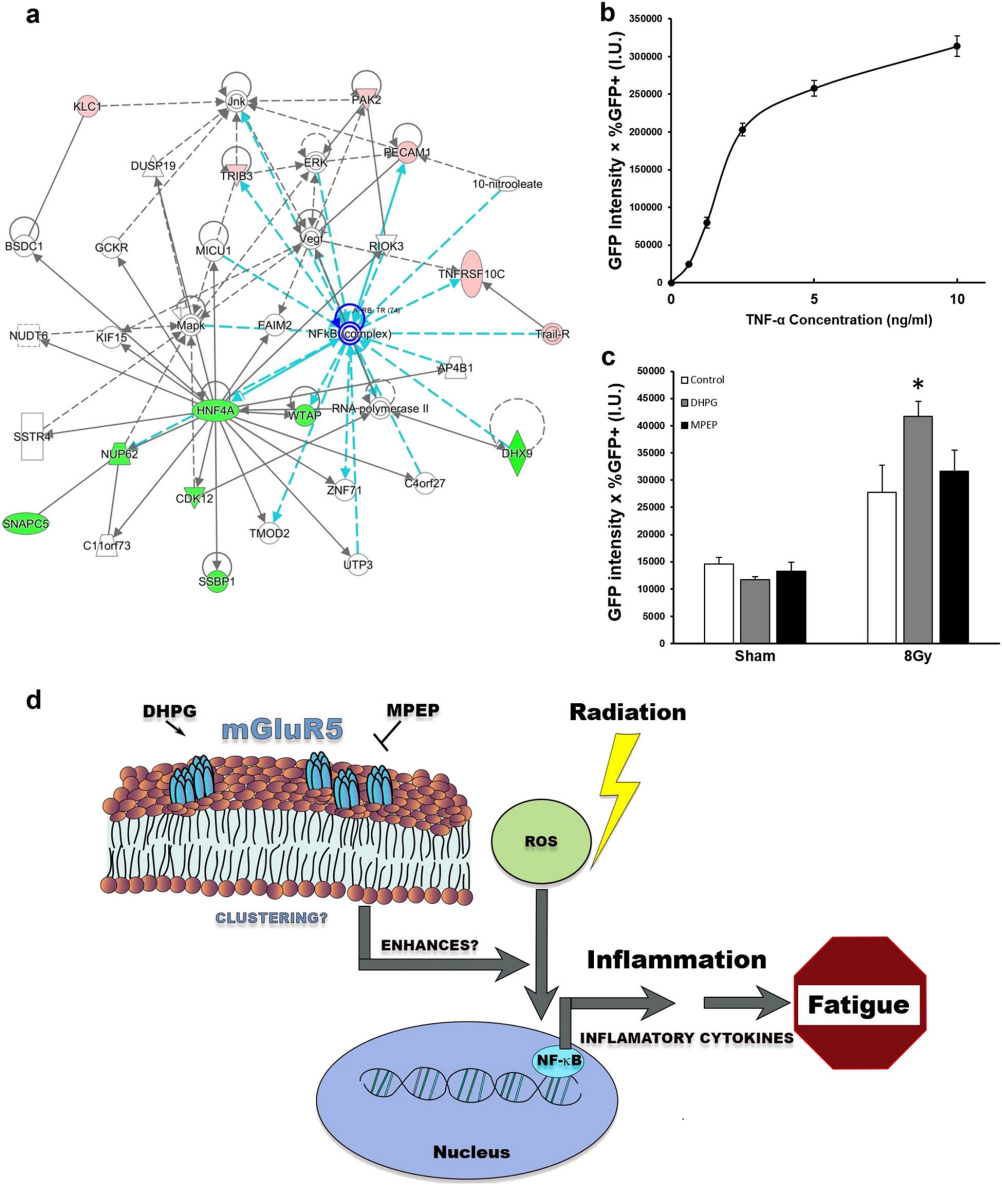
mGluR5 modulation enhanced the effect of irradiation on NF-κB activation. **a** Pathway analysis network containing differentially expressed genes from 1-year post radiotherapy identified around the *NFKB* gene. Red nodes indicate genes upregulated in fatigued subjects compared to non-fatigued subjects, green nodes indicate downregulated genes. **b** GFP intensity increased with increasing concentrations of TNFα in the NF-κB/GFP Jurkat reporter cell line. **c** Irradiation significantly increased NF-κB/GFP activation in the NF-κB/GFP Jurkat reporter cell line (*p* = 0.01). DHPG pretreatment enhanced the effect of irradiation on NF-κB activation (*p* = 0.04). Error bars = s.e.m. **d** A schematic illustration of the role of mGluR5 in post-radiotherapy fatigue in cancer patients. mGluR5 modulation amplified radiation-induced NF-κB activation, which predisposed cancer patients to post-radiotherapy inflammation and persistent fatigue

IPA interaction map analysis of the most differentially expressed genes identified using microarray data collected at 1-year post radiotherapy indicated that a commonality in the pathways was the NF-κB pathway (Fig. 4a). Further, IPA upstream analysis revealed NF-κB as a significant upstream regulator of differentially expressed genes in fatigued subjects (*p* = 0.00036). In fact, NF-κB target genes include RANTES and TRAIL, which we had previously shown to be related to fatigue 1-year post radiotherapy^6^. To investigate whether mGluR5 activation prior to irradiation modulated NF-κB activity, we employed a Jurkat reporter cell line that expresses GFP upon NF-κB activation. NF-κB/GFP reporter cells were first treated with increasing concentrations of recombinant TNFα, a potent NF-κB inducer, to establish that NF-κB activation led to GFP production in the reporter cell line (Fig. 4b). The NF-κB/GFP reporter cells were then treated with control, DHPG, or MPEP prior to irradiation at 8 Gy. Irradiation alone induced NF-κB activation compared to the sham irradiated cells. Pretreatment with DHPG further increased NF-κB activation compared to the irradiated control cells (*p* = 0.01), suggesting that mGluR5 activation amplified the effect of irradiation on NF-κB transcription activation (Fig. 4c).

## Discussion

Persistent fatigue is a common complaint that greatly affects the quality of life of cancer survivors^1^. Little is known about the underlying mechanism of fatigue, and no therapeutic intervention is currently available to address this debilitating disorder. Therefore, there is an urgent need to understand the etiology of persistent fatigue in cancer survivors to develop better treatment options. Previous studies point to an inflammatory etiology triggered by repeated stress such as oxidative stress from radiotherapy^6^. In the current study, we found a novel gene signature, “Glutamate Signaling Pathway,” that identified patients at risk for developing fatigue one year after radiotherapy. Transcripts of the predictive gene *GRM5* encoding mGluR5 were detectable in fatigued subjects at both baseline and 1-year post radiotherapy, but not in non-fatigued subjects. Consistent with previous literature^20^, mGluR5 was constitutively expressed in Jurkat cells, and pretreatment with DHPG prior to irradiation resulted in mGluR5 clustering. RANTES release by irradiated T cells was enhanced by DHPG pretreatment, whereas MPEP had the opposite effect. DHPG or MPEP treatment in the absence of irradiation did not affect RANTES release compared to control. These findings suggest that mGluR5 activation may prime T cells to irradiation-induced activation. Consistent with the inflammatory hypothesis, pretreatment with DHPG amplified radiation-induced NF-κB activation, suggesting a role of mGluR5 in modulating T cell activation after radiotherapy via NF-κB transcriptional activation (Fig. 4d). This hypothesis may help explain that baseline levels of mGluR5, as part of the glutamate receptor signaling pathway, predispose cancer patients to post-radiotherapy fatigue. To our knowledge, this is the first evidence of the involvement of mGluR5 in the pathogenic process of cancer-related fatigue.

Glutamate is the major excitatory neurotransmitter in the central nervous system (CNS), and signaling via glutamate receptors mediate essentially all facets of neurotransmission^21^. In recent years, the role of glutamate signaling has been found to extend beyond the CNS including playing a key role in immune regulation^22–24^. Glutamate has been shown to be released by various cells in the peripheral immune cells including T cells, neutrophils, dendritic cells, monocytes, and macrophages^25–29^. In addition, glutamate has been shown to play a direct role in regulating lymphocyte immune function and cytokine release^30^. Moreover, mGluRs including mGluR5 have been identified in a variety of immune cells including T lymphocytes^20,23^.

In our study, mGluR5 was found to be constitutively expressed in T cells and DHPG pretreatment resulted in cluster formation after irradiation. Although homodimerization is required for mGluRs to function, aberrant clustering of mGluR5 receptors in the brain has been shown to elevate intracellular calcium and synapse deterioration, which can be prevented by mGluR5 antagonist^31^. Long forms of Homer have been shown to induce cell surface clusters of mGluR5 in neurons due to the interaction with C-terminal tails of Homer, which brings the complex in association with IP3 enhancing intracellular calcium signaling^32^. It is possible that aberrant clustering of mGluR5 in the DHPG-irradiation group amplified downstream signaling of the receptor. mGluR5 activation via secondary messenger cAMP leads to nuclear translocation of NF-κB subunit p65, allowing the NF-κB dimer to translocate into the nucleus, where it turns on transcription of its target genes^33^. One of the downstream target genes expressed upon NF-κB transcriptional activation is the pro-inflammatory chemokine RANTES, which promotes chronic inflammation by recruiting and activating cells involved in inflammation such as monocytes, lymphocytes, eosinophils, and mast cells^34^. Interestingly, another NF-κB target gene TRAIL was previously shown to be upregulated in fatigued subjects 1-year post radiotherapy^6^. In addition to directly influencing the inflammation cascade, mGluRs may interact with other receptors to modulate the inflammatory tone. For example, although adenosine A_2A_ receptors (A_2A_-R) are considered anti-inflammatory, interactions between A_2A_-R and mGluR5 in lymphocytes can trigger proinflammatory responses via PLC-PKC signaling^35^.

Interestingly, treatment with DHPG and MPEP without irradiation did not affect mGluR5 clustering, RANTES release, or NF-κB transcription factor activation under experimental conditions in this study. The role of mGluR5 activation appeared to be enhancement of irradiation-induced increase in NF-κB activation and pro-inflammatory cytokine release. Indeed, baseline fatigue levels in subjects that developed fatigue 1-year post radiotherapy did not differ from non-fatigued subjects, even though mGluR5 mRNA transcripts were significantly higher in the fatigued subjects. It is possible that higher levels of mGluR5 alone in this patient population does not directly lead to fatigue. Instead, increased mGluR5 gene expression primes the immune system to develop chronic inflammation after the initial trigger of oxidative stress from radiotherapy, which eventually leads to a prolonged inflammatory state. Immune responses to stressors are normally tightly regulated, and mechanisms including activation-induced cell death (AICD) in T cells are employed to control inflammation^36^. Interestingly, glutamate has been shown to inhibit AICD in activated T cells via group I mGluRs^37^. It is possible that mGluR5 activation prior to irradiation results in sustained T cell activation and NF-κB transcription factor activation. By doing so, individuals with higher levels of mGluR5 are particularly vulnerable to developing chronic inflammation and fatigue after receiving cancer therapy.

An interesting observation in the current study is that MPEP treatment prior to irradiation decreased RANTES release (Fig. 3c) but did not significantly affect NF-κB activation (Fig. 4c). While MPEP is commonly used as a specific mGluR5 antagonist, previous studies have reported possible off-target effects of MPEP on other glutamate receptors, such as NMDA 1 A/2B and kainate Glu6-(IYQ)^38^. Although much remains unknown about the role of glutamate receptors in immune signaling, it is possible that MPEP modulation of other glutamate receptors may exert additional influence on NF-κB activation in T lymphocytes^24,39,40^. Further contributing to the complexity of RANTES transcriptional control, the human RANTES/CCL5 promoter region appears to contain binding sites for multiple transcription factors including NF-κB, AP-1, C/EBP, and Ets-1^41–43^. It is possible that MPEP pre-treatment influences RANTES release via multiple transcription factors including NF-κB. Future studies will investigate the relationship between MPEP, RANTES, and other transcription factors that may be involved.

The acute-phase inflammatory marker, CRP, has been shown in previous studies as a predictive marker for post-treatment survival in various malignancies including prostate cancer^44,45^. In the current study, plasma CRP levels between fatigued and non-fatigued subjects were significantly different at baseline, but not at 1-year post-radiotherapy. Interestingly, baseline CRP significantly correlated with fatigue symptom severity 1-year post-radiotherapy, suggesting that CRP may serve as a predictive marker for both post-treatment survival, as well as fatigue. It is possible that CRP and mGluR5 may be mediators of independent inflammatory events, as no significant correlation between mGluR5 transcript levels and baseline CRP concentrations was observed. Furthermore, CRP is a non-specific marker of systemic inflammation, so it is not clear whether the elevated baseline CRP levels in fatigued subjects was secondary to other causes of inflammation, which include cancer itself. Even though all subjects recruited in the current study were diagnosed with non-metastatic prostate cancer before undergoing radiotherapy, it is possible that subtle differences in baseline inflammatory tone, perhaps related to cancer severity, were further exacerbated by mGluR5-related downstream events in response to radiotherapy leading to persistent fatigue 1-year post-radiotherapy. Future studies will explore the interplay between baseline inflammation, as well as radiotherapy-induced inflammatory events in fatigue outcomes in a larger sample size.

It is worth pointing out that this study only investigated the role of mGluR5 in irradiation-induced activation in T lymphocytes. It is possible that immune cells other than T lymphocytes contributed to higher levels of *GRM5* transcripts in fatigued subjects. Although many individual differences may help explain the variability in response to the same stressor, T cells have been well studied because of their pivotal role in orchestrating the immune response^46^. As the most important cells in adaptive immunity, T cells are often implicated in initiating the inflammatory cascade^47,48^. Therefore, we opted to investigate the role of mGluR5 in T cell activation. Future studies will further explore the contribution to persistent fatigue by other mGluR5-expressing immune cells. Another limitation of the study is that we only investigated T cell activation after global mGluR5 activity modulation, regardless of the location of the receptor. While the role of mGluR5 in immune regulation is still emerging, studies on neuronal mGluR5 demonstrated that intracellular and cell surface mGluR5 regulate transcriptional activation and gene expression differently^49,50^. Although it is beyond the scope of the current study, future studies will investigate whether the role of mGluR5 in fatigue pathogenesis is related to the activities of intracellular mGluR5 or cell surface receptors influencing behavior by altering immune response to stressors.

Repeated stress in the form of ionizing radiation from radiotherapy triggers different long-term responses in different patients, as demonstrated by the observation that only a subset of patients develop chronic inflammation and persistent fatigue^6^. Accordingly, determining the genetic susceptibility that leads to unresolved inflammation is crucial to understanding the underpinnings of fatigue pathogenesis. Genetic vulnerability combined with cancer and old age predispose cancer patients to developing chronic inflammatory conditions after receiving repeated stress. We demonstrated that mGluR5 activation prior to irradiation may lead to sustained T cell activation and NF-κB transcription factor activation. Our study demonstrates for the first time that glutamate receptor signaling predicts the development of fatigue long after treatment completion. The novel finding in this study will not only lead to a greater understanding of fatigue and other symptoms related to chronic inflammation, it will also provide potential therapeutic targets to treat this debilitating condition.

## Electronic supplementary material

Supplementary Table 1
