# Supplementary material for: mGluR5 mediates post-radiotherapy fatigue development in cancer patients
Source: Transl Psychiatry. 2018 May 30;8:110. doi: 10.1038/s41398-018-0161-3 (PMC5976668; doi:10.1038/s41398-018-0161-3)
Supplement: Supplementary file 1 — Supplementary Table 1 [file 41398_2018_161_MOESM1_ESM.docx]

**Supplementary Table 1**. Complete list of the 420 genes generated using the feature selection procedure.

**Probe Name Gene Name Fold change Fisher's Ratio**

203290_at HLA-DQA1 1.13 1.3

203290_at HLA-DQA1 0.98 0.83

203290_at HLA-DQA1 0.95 1.61

203290_at HLA-DQA1 0.8 0.84

209710_at GATA2 0.71 0.71

205624_at CPA3 0.69 0.79

205624_at CPA3 0.66 0.97

205624_at CPA3 0.63 1

217736_s_at EIF2AK1 0.55 0.71

205624_at CPA3 0.51 0.83

216054_x_at MYL4 0.45 0.71

1564467_at FAM161A 0.42 1.02

210358_x_at GATA2 0.42 0.97

237021_at CCDC41-AS1 0.36 0.78

235723_at BNC2 0.35 0.73

207464_at AHCYL1 0.34 1.03

1554547_at FAM13C 0.34 0.93

230068_s_at PEG3-AS1 0.34 0.76

235572_at SPC24 0.34 0.72

235616_at TSHZ2 0.34 0.7

237656_at WWC2 0.33 0.79

235915_at --- 0.33 0.79

207503_at TCP10 0.33 0.77

231081_at C2orf73 0.33 0.74

241805_at GABRG1 0.33 0.74

212127_at RANGAP1 0.33 0.73

242587_at SLC9A9 0.33 0.72

1566836_at MRPL49P1 /// MRPL49P1 0.33 0.72

1552414_at WFDC9 0.33 0.72

231206_at --- 0.33 0.7

1560643_x_at --- 0.32 0.83

1553207_at ARL10 0.32 0.82

234010_at --- 0.32 0.76

1557753_at OTTHUMG00000161199 /// RP11-519M16.1 0.32 0.73

1565722_at LOC100128281 0.31 0.96

1557758_at OTTHUMG00000166294 /// RP11-680E19.1 0.31 0.82

229365_at PPP1R3F 0.31 0.78

217157_x_at IGK /// IGKC 0.31 0.71

205624_at CPA3 0.31 0.7

240055_at --- 0.31 0.7

214634_at HIST1H4A /// HIST1H4B /// HIST1H4C /// HIST1H4D /// HIST1H4E /// HIST1H4F /// HIST1H4H /// HIST1H4I /// HIST1H4J /// HIST1H4K /// HIST1H4L /// HIST2H4A /// HIST2H4B /// HIST4H4 0.3 0.9

226702_at CMPK2 0.3 0.79

238362_at --- 0.3 0.78

214769_at CLCN4 0.3 0.77

224362_at LOC100128922 0.3 0.75

207627_s_at TFCP2 0.3 0.74

225491_at SLC1A2 0.29 1.31

239459_s_at CYP19A1 /// LOC100652954 0.29 1.15

234323_at THSD4 0.29 1.05

236239_at XPNPEP1 0.29 0.89

220912_at --- 0.29 0.84

228376_at GGTA1P 0.29 0.83

239288_at TNIK 0.29 0.78

217412_at TRAV9-2 /// TRAV9-2 0.29 0.71

214796_at KIAA1456 0.28 0.87

244191_at RPLP1 0.28 0.83

234623_x_at --- 0.28 0.83

208218_s_at ACVR1B 0.28 0.82

238006_at SIN3A 0.28 0.81

214217_at GRM5 0.28 0.8

206889_at PDIA2 0.28 0.79

1568661_at --- 0.28 0.78

243973_at --- 0.28 0.77

1569499_at --- 0.28 0.74

232659_at --- 0.28 0.72

233388_at --- 0.28 0.72

224911_s_at DCBLD2 0.28 0.71

239768_x_at GPATCH2 0.27 1.12

1556247_a_at LOC100506271 0.27 0.91

1562627_at LOC100130078 0.27 0.87

227226_at MRAP2 0.27 0.86

216665_s_at TTTY2 /// TTTY2B 0.27 0.85

236676_at --- 0.27 0.81

226709_at ROBO2 0.27 0.8

214826_at PDE12 0.27 0.79

1557551_at --- 0.27 0.77

1562510_at LOC339442 0.27 0.77

238633_at EPC1 0.27 0.77

237355_at --- 0.27 0.75

1570480_s_at ART1 0.27 0.75

206606_at LIPC 0.27 0.74

244573_at --- 0.27 0.73

215868_x_at --- 0.27 0.73

241257_at --- 0.27 0.72

1559293_x_at LINC00032 0.27 0.71

205613_at SYT17 0.27 0.7

1567256_at OR1J2 0.26 1.27

215074_at MYO1B 0.26 1.2

1555931_at TSGA10 0.26 1.12

214071_at GNAL 0.26 1.1

1563898_at OTTHUMG00000169268 /// RP11-421F16.3 0.26 0.99

1552745_at SLCO6A1 0.26 0.94

203945_at ARG2 0.26 0.94

243184_at --- 0.26 0.89

224320_s_at MCM8 0.26 0.86

240144_at DNASE1 0.26 0.84

243438_at PDE7B 0.26 0.82

239498_at OTTHUMG00000183933 /// RP11-498E2.7 0.26 0.82

240544_at --- 0.26 0.78

205799_s_at SLC3A1 0.26 0.74

204338_s_at RGS4 0.26 0.74

216426_at TCEB1P3 /// TCEB1P3 0.26 0.72

1561635_at --- 0.26 0.72

219436_s_at EMCN 0.26 0.72

61734_at RCN3 0.26 0.72

231129_at LOC728012 0.26 0.72

210747_at HLA-DQB1 0.26 0.71

206013_s_at ACTL6B 0.26 0.7

240879_x_at --- 0.26 0.7

1560855_at --- 0.26 0.7

1557217_a_at FANCB 0.25 1.01

206937_at SPTA1 0.25 1

236823_at IDS 0.25 0.91

1559839_at TBX18 0.25 0.88

1560901_at --- 0.25 0.87

1568904_at OTTHUMG00000175990 /// RP11-218F4.1 0.25 0.83

207699_at ZFHX2 0.25 0.82

206786_at HTN3 0.25 0.81

1553003_at PKHD1 0.25 0.81

1564125_at LOC285857 0.25 0.81

223753_s_at CFC1 /// CFC1B 0.25 0.8

213468_at ERCC2 0.25 0.8

214580_x_at KRT6A /// KRT6B /// KRT6C 0.25 0.79

206509_at PIP 0.25 0.77

239689_at --- 0.25 0.77

232437_at CPSF3L 0.25 0.76

1561593_at OTTHUMG00000161471 /// RP11-400D2.2 0.25 0.76

220821_at GALR1 0.25 0.72

222328_x_at MEG3 0.25 0.72

231023_at CARS2 0.25 0.71

1563638_at TVP23A 0.25 0.71

1556289_at LOC100506016 0.24 1.03

1560253_at LHX9 0.24 1

223515_s_at COQ3 0.24 0.98

233602_at OTTHUMG00000176170 /// RP11-95O2.1 0.24 0.89

238891_at --- 0.24 0.87

205543_at HSPA4L 0.24 0.86

225599_s_at TRIQK 0.24 0.85

1555375_at GRIK2 0.24 0.8

1554034_a_at FRMD4A 0.24 0.8

1556233_s_at KIF6 0.24 0.79

220034_at IRAK3 0.24 0.79

1561054_a_at CCDC14 0.24 0.76

1559075_s_at BAHCC1 0.24 0.76

1568850_at CCBP2 0.24 0.76

1568711_a_at LOC100507498 0.24 0.73

210593_at SAT1 /// ZSWIM8 0.24 0.73

236400_at IDH1-AS1 0.24 0.72

205164_at GCAT 0.24 0.72

1565778_at ABCA8 0.24 0.71

228005_at ZXDB 0.24 0.71

1553296_at GPR128 0.24 0.7

1566267_at --- 0.23 1.19

208247_at ERC2-IT1 0.23 1.08

1553157_at LHX4 0.23 1.05

213356_x_at HNRNPA1 /// HNRNPA1L2 /// HNRNPA1P10 /// HNRNPA1P33 0.23 0.99

237586_at --- 0.23 0.88

213894_at THSD7A 0.23 0.86

237228_at ZDHHC1 0.23 0.86

207496_at MS4A2 0.23 0.85

231692_at PIGG 0.23 0.83

216255_s_at GRM8 0.23 0.82

216886_at CHRNA4 0.23 0.8

231309_at LOC100996298 0.23 0.78

241331_at SKAP2 0.23 0.78

220878_at --- 0.23 0.76

213257_at SARM1 0.23 0.75

204450_x_at APOA1 0.23 0.75

242458_at RALGPS2 0.23 0.75

240700_at TOR1AIP2 0.23 0.74

1568981_at --- 0.23 0.72

202019_s_at LANCL1 0.23 0.71

203441_s_at CDH2 0.23 0 .71

1560161_at OTTHUMG00000171943 /// RP11-59H7.3 0.23 0.7

242022_at --- 0.23 0.7

237240_at --- 0.23 0.7

217904_s_at BACE1 0.23 0.7

1552430_at WDR17 0.23 0.7

237505_at --- 0.22 1.72

1557876_at LOC340094 0.22 1.13

1560491_at CTC-340A15.2 /// OTTHUMG00000162877 0.22 1.08

220574_at SEMA6D 0.22 1.03

233522_at CTC-454M9.1 /// OTTHUMG00000162630 0.22 0.96

242619_x_at --- 0.22 0.9

231499_s_at --- 0.22 0.88

215621_s_at IGHD 0.22 0.88

1552904_at NETO1 0.22 0.87

235691_at LOC729970 0.22 0.86

223395_at ABI3BP 0.22 0.85

1569040_s_at ANKRD36BP2 /// LOC101060554 0.22 0.84

207051_at SLC17A4 0.22 0.84

214546_s_at P2RY11 /// PPAN-P2RY11 0.22 0.83

211438_at TRHR 0.22 0.83

1554618_at AGFG2 0.22 0.81

207854_at GYPE 0.22 0.8

207367_at ATP12A 0.22 0.8

1568919_at OTTHUMG00000020399 /// RP11-341A22.2 0.22 0.8

227623_at CACNA2D1 0.22 0.78

237148_at --- 0.22 0.77

1562274_at OTTHUMG00000176637 /// RP11-22H5.2 0.22 0.77

233067_at PRDM11 0.22 0.76

244866_at --- 0.22 0.76

243506_at C18orf42 0.22 0.75

236628_at --- 0.22 0.74

242387_at TDRP 0.22 0.74

244135_at OTTHUMG00000163822 /// RP11-177H2.2 0.22 0.73

240390_at GALNT5 0.22 0.71

1555610_at AGK 0.22 0.71

234857_at --- 0.22 0.7

205503_at PTPN14 0.22 0.7

1554703_at ARHGEF10 0.21 1.21

207517_at LAMC2 0.21 0.91

231675_s_at ADH4 0.21 0.89

242740_at --- 0.21 0.85

233956_at --- 0.21 0.84

227300_at TMEM119 0.21 0.83

1557583_at ST18 0.21 0.83

215357_s_at POLDIP3 0.21 0.8

222081_at OTTHUMG00000175899 /// RP1-223E5.4 0.21 0.79

1570049_at LINC00910 0.21 0.79

240470_at --- 0.21 0.77

1559762_at --- 0.21 0.77

243368_at --- 0.21 0.76

223669_at HEMGN 0.21 0.75

1561091_at OTTHUMG00000157300 /// RP11-255G21.1 0.21 0.73

52255_s_at COL5A3 0.21 0.73

214532_x_at POU5F1B 0.21 0.71

1562562_at ANKUB1 0.21 0.71

214142_at ZG16 0.21 0.71

243427_at --- 0.21 0.7

1552999_a_at WFDC10B 0.21 0.7

236095_at NTRK2 0.21 0.7

1565723_at LOC100128281 0.2 1.24

224067_at --- 0.2 1.07

205064_at SPRR1B 0.2 1.02

221157_s_at FBXO24 0.2 1

1568682_a_at LOC100507336 0.2 0.96

1565838_at --- 0.2 0.91

237243_at --- 0.2 0.86

241009_at FOXN4 0.2 0.86

243743_at --- 0.2 0.83

1569024_at FAM13A 0.2 0.81

1564672_at --- 0.2 0.81

231971_at FANCM 0.2 0.8

244027_at --- 0.2 0.79

240556_at DCN 0.2 0.79

220552_at TRPC5 0.2 0.75

232542_at COL9A2 0.2 0.75

239090_at FAM161A 0.2 0.74

1560642_at --- 0.2 0.73

1565027_at OFCC1 0.2 0.73

1569843_at DYNC1I1 0.2 0.73

229698_at SHANK3 0.2 0.73

205673_s_at ASB9 0.2 0.72

1552698_at TUBA3FP 0.2 0.72

230250_at PTPRB 0.2 0.71

230562_at LOC100507530 0.2 0.71

222247_at --- 0.2 0.71

216982_x_at --- 0.2 0.7

217264_s_at SCNN1A 0.2 0.7

1560063_a_at CTD-2540F13.2 /// OTTHUMG00000182526 0.19 1.16

231180_at --- 0.19 1.11

232853_at OTTHUMG00000176034 /// RP11-552M11.8 0.19 1.05

1559103_s_at OTTHUMG00000176315 /// RP11-73K9.2 0.19 0.94

205440_s_at NPY1R 0.19 0.93

216469_at LOC441666 0.19 0.88

233205_at --- 0.19 0.87

229696_at FECH 0.19 0.86

233755_at OTTHUMG00000164543 /// RP11-758M4.4 0.19 0.86

237543_at LOC100506286 0.19 0.85

241295_at --- 0.19 0.84

1570650_at CCBL1 0.19 0.82

1555313_a_at MCF2 0.19 0.81

211885_x_at FUT6 0.19 0.8

1559228_at CLU 0.19 0.79

211552_s_at ALDH4A1 0.19 0.79

235905_at ZNF704 0.19 0.79

233098_s_at EPB41L4B 0.19 0.77

220002_at KIF26B 0.19 0.76

1553209_at RNFT2 0.19 0.75

1555942_a_at MIR205HG /// MIR205HG 0.19 0.74

207518_at DGKE 0.19 0.73

217275_at TSSK2 0.19 0.73

217511_at KAZALD1 0.19 0.73

229049_at LOC100506655 0.19 0.73

230258_at GLIS3 0.19 0.73

1553188_s_at PARD3B 0.19 0.73

1569540_at --- 0.19 0.72

1559680_at TTL 0.19 0.72

205775_at FAM50B 0.19 0.72

210037_s_at NOS2 0.19 0.71

210806_at TTLL5 0.19 0.71

241220_at --- 0.19 0.7

1570320_at --- 0.18 1.33

1559848_at NSUN4 0.18 1.14

206528_at TRPC6 0.18 1.03

202489_s_at FXYD3 0.18 0.96

1552975_x_at --- 0.18 0.94

241453_at PTK2 0.18 0.9

209908_s_at TGFB2 0.18 0.88

212594_at MIR4680 /// PDCD4 0.18 0.88

1556960_a_at ANHX 0.18 0.83

220228_at AP4E1 0.18 0.8

222010_at SNORA29 /// TCP1 0.18 0.79

1562699_at OTTHUMG00000167662 /// OTTHUMG00000181593 /// RP11-629G13.1 0.18 0.79

220324_at LINC00472 0.18 0.78

1557791_at OTTHUMG00000172754 /// RP4-676L2.1 0.18 0.78

229361_at SLC25A27 0.18 0.77

1555529_at RNH1 0.18 0.76

1567878_at DEFB114 0.18 0.76

212398_at RDX 0.18 0.76

228739_at CYS1 0.18 0.75

31835_at HRG 0.18 0.75

225895_at SYNPO2 0.18 0.75

1556786_at PDE5A 0.18 0.75

216359_at MUC7 0.18 0.73

243059_at FENDRR 0.18 0.72

244500_s_at EVI5L 0.18 0.72

217568_at EDDM3A 0.18 0.72

1562693_at OTTHUMG00000031842 /// RP5-971N18.3 0.18 0.71

234587_at --- 0.18 0.71

243741_at --- 0.18 0.7

231117_at FAM181A 0.17 1.04

1555412_at FBXL21 0.17 1.02

1562991_at ZNF292 0.17 1

208256_at EFNA2 0.17 0.98

1561136_at GYPE 0.17 0.98

205912_at PNLIP 0.17 0.89

216466_at NAV3 0.17 0.86

228307_at EMILIN3 0.17 0.83

206721_at CCDC181 0.17 0.79

1558703_at SLC46A1 0.17 0.79

236597_at UGT3A1 0.17 0.79

214914_at FAM13C 0.17 0.78

244209_at LBX1-AS1 0.17 0.77

214515_at OR1E1 0.17 0.77

204699_s_at DIEXF 0.17 0.76

241029_at --- 0.17 0.76

214380_at PRPF31 0.17 0.74

1555214_a_at CLEC7A 0.17 0.73

216841_s_at LOC100129518 /// SOD20.17 0.73

229462_at --- 0.17 0.71

207302_at SGCG 0.17 0.71

244355_at AVL9 0.17 0.7

215066_at PTPRF 0.17 0.7

233026_s_at PDZD2 0.17 0.7

1561488_at CTA-331P3.1 /// OTTHUMG00000175938 0.17 0.7

228724_at TTLL7 0.17 0.7

206132_at MCC 0.16 1

222911_s_at CXorf36 0.16 0.94

210575_at NUDC 0.16 0.92

231817_at USP53 0.16 0.92

1563709_at LOC100127955 /// LOC100128374 0.16 0.89

232404_at SHROOM4 0.16 0.81

1561988_at LOC286068 0.16 0.81

233944_at --- 0.16 0.8

1555396_s_at CXorf67 0.16 0.8

233744_at --- 0.16 0.79

210850_s_at ELK1 0.16 0.79

1560570_a_at OTTHUMG00000175734 /// RP11-111J6.2 0.16 0.76

223800_s_at LIMS3 /// LIMS3L 0.16 0.73

208567_s_at KCNJ12 /// KCNJ18 /// LOC100996843 0.16 0.72

1552826_at SLC26A7 0.16 0.72

1569741_at OTTHUMG00000176177 /// RP11-653G8.2 0.16 0.71

230784_at PRAC 0.15 0.88

238772_at ZNF207 0.15 0.83

223783_s_at DBIL5P 0.15 0.81

237885_at SOX21-AS1 0.15 0.8

237690_at GPR115 0.15 0.76

1560184_at TMCO5A 0.15 0.76

238325_s_at ODF3B 0.15 0.74

231060_at --- 0.15 0.73

1560416_at DNAH11 0.15 0.72

214230_at CDC42 0.15 0.72

227429_at EFCAB4A 0.15 0.71

1561106_at LINC00271 0.15 0.7

1561728_a_at LINC00642 0.14 0.94

220175_s_at CBWD1 /// CBWD2 /// CBWD3 /// CBWD5 /// CBWD6 /// CBWD7 /// LOC100653334 /// LOC101060578 0.14 0.8

240192_at GATA3-AS1 0.14 0.79

1554512_a_at CEP89 0.14 0.78

211046_at KCNH6 0.14 0.77

235015_at ZDHHC9 0.14 0.76

1553822_at RTP1 0.14 0.74

215930_s_at CTAGE5 0.14 0.73

243324_x_at TNPO1 0.14 0.72

203875_at SMARCA1 0.13 1.08

235819_at BTF3L4 0.13 0.9

239537_at ST8SIA2 0.13 0.89

234804_at PROX2 0.13 0.83

1558897_at PLK5 0.13 0.8

235591_at SSTR1 0.13 0.76

230731_x_at ZDHHC8 0.13 0.74

224402_s_at FCRL4 0.13 0.73

218275_at SLC25A10 0.13 0.72

219564_at KCNJ16 0.13 0.71

211305_x_at FCAR 0.13 0.7

201712_s_at RANBP2 0.13 0.7

235955_at MARVELD2 0.12 0.97

241032_at ANKRD40 0.12 0.82

243760_at MIPEPP3 0.12 0.75

241412_at BTC 0.11 0.85

215345_x_at TRGV7 /// TRGV7 0.11 0.74

221470_s_at IL37 0.11 0.74

1559956_at SYT7 0.11 0.7

206952_at G6PC 0.1 0.87

1555375_at GRIK2 0.09 0.72

238962_at ZNF681 -0.19 1.04

218151_x_at SLC52A2 -0.21 0.77

234726_s_at TMEM168 -0.22 0.71

217790_s_at SSR3 -0.25 0.84

225677_at BCAP29 -0.28 0.76

205046_at CENPE -0.3 0.91

234978_at SLC36A4 -0.36 0.7

235005_at DIS3L -0.39 0.7

215242_at LOC100505991 /// PIGC -0.45 0.72

213915_at NKG7 -0.47 0.88

223543_at PDZD4 -0.48 0.72

215242_at LOC100505991 /// PIGC -0.6 0.91

202239_at PARP4 -0.74 0.82

215242_at LOC100505991 /// PIGC -0.92 0.91
